# Lipidation of NOD2 Agonists with Adamantane and Stearoyl Moieties Differentially Regulates Their In Vivo Adjuvant Activity

**DOI:** 10.3390/pharmaceutics14122755

**Published:** 2022-12-09

**Authors:** Samo Guzelj, Marcela Šišić, Špela Bizjak, Leo Frkanec, Ruža Frkanec, Žiga Jakopin

**Affiliations:** 1 Faculty of Pharmacy, University of Ljubljana, SI-1000 Ljubljana, Slovenia; 2 Centre for Research and Knowledge Transfer in Biotechnology, University of Zagreb, 10000 Zagreb, Croatia; 3 Rudjer Bošković Institute, 10000 Zagreb, Croatia

**Keywords:** NOD2 agonist, vaccine adjuvant, adamantane, immunopotentiators, desmuramylpeptides

## Abstract

NOD2 is an innate immune receptor that constitutes an important target for the development of small molecule immunopotentiators with great potential to be used as vaccine adjuvants. We report here the results of an in vivo study of the adjuvant properties of a desmuramylpeptide NOD2 agonist SG29 and its lipidated analogs featuring an adamantyl moiety or a stearoyl group. These compounds have been synthesized, incorporated into liposomes, and evaluated for their in vivo adjuvant activity. The characterization of liposome formulations of examined compounds revealed that their size increased in comparison to that of empty liposomes. The introduction of a stearoyl or an adamantane lipophilic anchor into the structure of SG29, to produce SG115 and ZSB63, respectively, substantially improved the in vivo adjuvant activity. Of note, the attachment of the stearoyl moiety produced a Th2-biased immune response, while the incorporation of the adamantyl moiety greatly enhanced the production of total IgG but mostly augmented the production of IgG2a antibodies, which indicated a shift toward a Th1 immune response. The identified bona fide capacity of ZSB63 to initiate a cellular immune response thus highlights its untapped potential as an alternative vaccine adjuvant.

## 1. Introduction

Nucleotide-binding oligomerization domain-containing protein 2 (NOD2) is a cytoplasmic pattern-recognition receptor widely expressed in immune cells. It is activated by fragments of bacterial peptidoglycan resembling muramyl dipeptide (MDP), which results in pro-inflammatory cytokine production, type I interferon secretion, and expression of co-stimulatory molecules [1]. NOD2 activation also triggers the maturation and activation of antigen-presenting cells (APCs) [2], which proved to be an essential and highly desirable trait for vaccine adjuvant development [3,4]. During the past decade, we have been heavily engaged in the design and synthesis of desmuramylpeptide NOD2 agonists [5,6,7], which culminated in the discovery of a nanomolar NOD2 agonist SG29 (EC50, 45 ± 6 nM) [8]. Next-generation adjuvants typically include rationally designed synthetic immune potentiators that are appropriately formulated [9,10,11]. Liposomes with built-in immunostimulants represent potent adjuvant formulations that act as a depot at the injection site, ensure the protection of antigens from the action of the hydrolytic enzymes, and, most importantly, enhance the production of specific cytokines and strengthen the overall immune response [12,13]. Liposomes’ size, surface charge, and composition can greatly affect the efficiency of liposomes as drug delivery systems [14,15,16]. Due to their particulate structure, they are phagocytized by APCs, thus facilitating intracellular delivery. Of note, liposomes were also recognized as promising carriers for NOD2 agonists [7,17]. Surprisingly, while SG29 demonstrated pronounced in vitro immuno-stimulating properties, i.e., the capacity to amplify the cytokine production in PBMCs and to augment dendritic cell-mediated activation of T cells, its liposomal formulation was devoid of in vivo adjuvant activity. 

Lipidation is a well-known approach to improve the intracellular delivery of compounds as well as facilitate their incorporation into liposomes. It is also worth mentioning that membrane targeting has recently been suggested as playing an important role in NOD2 activation by small molecules [18]. Amphiphilic molecules of adamantane as an auxiliary group has been exploited to facilitate interaction with the membrane, increase the cellular uptake of small peptides [19,20], and to enhance the adjuvant activity [21,22]. Further, decoration of NOD2 agonists with branched/linear fatty acids also led to noteworthy adjuvant activity, with muramyl tripeptide phosphatidylethanolamine (mifamurtide; MTP-PE), B30-MDP, and romurtide (MDP-Lys(L18)) being the most prominent lipophilic derivatives [23]. Similarly, the introduction of a C18 lipophilic anchor into the structure of in-house NOD2 agonist SG8 substantially improved the in vivo adjuvant activity to model antigen ovalbumin (OVA) [8]. These successful attempts prompted us to utilize a similar approach for the pharmacokinetic optimization of SG29. 

The aim of this study was to ascertain the effect of the installment of specific lipophilic substituents, namely adamantane and stearic acid, into the parent structure of SG29 on (i) the capacity to incorporate into liposomes and alter their size and charge, as well as (ii) the in vivo adjuvant activity. To that end, we generated a focused, small library of SG29 analogs with altered pharmacokinetic properties and determined their NOD2 agonistic activities in vitro in the HEK-Blue NOD2 cell line. Of those analogs, compound ZSB63 (EC50, 44 ± 9 nM) features an adamantane moiety installed via a short linker onto the phenol OH group of SG29 [24], while in compound SG115 (EC50, 6.2 ± 1.2 µM) the stearoyl group is directly attached to the aforementioned phenol group (see Table 1) [8]. These compounds allow for a direct comparison between the adamantyl and stearoyl moiety introduction and were therefore selected for further studies along with the parent compound SG29. The modified thin-lipid film method [25,26] was used to prepare the liposomal formulations of SG29, ZSB63, and SG115. Next, the liposomal formulations were characterized by dynamic light scattering (DLS) in terms of size distribution, polydispersity index, and zeta potential. Finally, they were evaluated for their adjuvant potential in a well-defined mouse model in conjunction with a model antigen ovalbumin. 

Their effect on the induction of a humoral immune response specific for OVA and their capacity to skew the immune response towards Th1/Th2 was interrogated by quantifying antigen-specific antibodies in the collected sera using ELISA methodology.

## 2. Materials and Methods

### 2.1. Materials, Antigens, and Antibodies

Muramyl dipeptide (MDP) was obtained from Invivogen, Inc., (San Diego, CA, USA). The lipidated NOD2 agonists (SG29, SG115, and ZSB63) were synthesized as described [8,24]. The HPLC purity of all pharmacologically investigated compounds was >95%. Bovine serum albumin, Tween 20, monoclonal anti-chicken egg albumin (clone OVA-14 mouse IgG1 isotype), and *o*-phenylenediaminedihydrochloride were obtained from Sigma (St. Louis, MO, USA). Horseradish-peroxidase-conjugated goat anti-mouse IgG (HRP-anti-mouse IgG) was obtained from Bio-Rad Laboratories (Hercules, CA, USA). Monoclonal antibodies (biotin-conjugated rat anti-mouse IgG1 and anti-mouse IgG2a) and streptavidin-HRP conjugate were purchased from PharMingen, Becton Dickinson (Franklin Lakes, NJ, USA). Chemicals used for the preparation of solutions and buffers were purchased from Kemika (Zagreb, Croatia). Ovalbumin (OVA) was purchased from Serva (Heidelberg, Germany). Egg-phosphatidylcholine (L-α-Phosphatidylcholine, type XI-E) was purchased from Avanti Polar Lipids. Cholesterol from the porcine liver was purchased from Sigma (USA).

### 2.2. Preparation of Liposomes

Multilamellar neutral liposomes were prepared by the modified thin-lipid film method [25,26]. Egg-phosphatidylcholine and cholesterol (total mass of lipid was 4 mg/mL in a molar ratio of 7:5) were dissolved in chloroform/methanol (volume ratio 2:1). The compounds SG29, SG115, and ZSB63 were incorporated into the liposomes by the modified film method. Namely, the compounds were dissolved in organic solvents and added to the chloroform/methanol solution of phospholipids. After rotary evaporation of the solvent, the remaining lipid film was dispersed in 1 mL of saline by vortexing. The concentration of compounds SG29, SG115, and ZSB63 was 3 mM. The liposome suspensions were stored at 4–8 °C overnight in order to swell and stabilize. Sequential extrusion using 0.5 mL extruder, through polycarbonate membranes with a pore diameter of 800 and 400 nm, was employed for reducing the size of the liposomes (LiposoFast; Avestin Inc., Ottawa, ON, Canada). Liposome suspensions, without a separation of non-entrapped material, were used for measurements of the physicochemical properties of liposomes. The preparation of liposome formulations of examined compounds for in vivo testing was slightly different. Namely, a dry lipid film was rehydrated with ovalbumin solution in a saline (0.1 mg/mL).

### 2.3. Dynamic Light Scattering Measurement (DLS)

The particle size distribution and zeta potential of the prepared liposomes were measured employing a Zetasizer Nano US (Malvern, UK) equipped with a green laser (532 nm) as described previously [27]. The intensity of scattered light was detected at an angle of 173°. Measurements were conducted at 25 °C. Obtained data were processed by the Zetasizer software 6.20 (Malvern Instruments). Each liposome suspension (200 µL) was diluted with saline solution (1.8 mL). The size of the liposomes is expressed as an average diameter (z-average) obtained from the Zetasizer Nano software, which calculated the size of liposomes from the signal intensity. Each sample was measured six times and the results were expressed as the average value. For the zeta potential measurements, liposomes were placed in special plastic cuvettes with a golden wire provided for the zeta potential determination. Each sample was measured three times and the results were expressed as the average value.

### 2.4. Mice

The NIH/OlaHsd inbred mice used in experiments were females, 2.0 to 2.5 months old, bred at the Institute of Immunology (Croatia). For the duration of the experiment, the mice were kept in the Animal Facility of the Institute of Immunology with unlimited access to food and water. The experiment was conducted in accordance with the Croatian Law on Animal Welfare (2017), which strictly complies with the EC Directive (2010/63/EU).

### 2.5. Immunizations

NIH/OlaHsd mice (five per group) were immunized subcutaneously in the tail base and boosted twice 21 days apart. The injection volume in all experimental groups was 0.1 mL per mouse, which corresponds to 10 μg of OVA, 400 μg of lipids, and 0.30 μmol of MDP, SG29, and lipidated SG29 analogs. The mice were anesthetized with i.p. application of ketamine/xylazine (25 mg/kg each) prior to blood collection from the axillary plexus, on the seventh day after the last booster dose. Individual sera from each animal were decomplemented at 56 °C for 30 min and then stored at −20 °C until tested.

### 2.6. Ovalbumin-Specific Serum Antibody Concentration Determination by ELISA

The levels of the OVA-specific total IgG, IgG1, and IgG2a in mice sera were determined by ELISA as detailed previously [7]. Briefly, flat-bottomed high-binding microtiter plates (Costar, New York, NY, USA) were coated with 1.5 mg/mL OVA solution in carbonate buffer, pH 9.6, and left overnight at room temperature (RT). Plates were washed and non-specific antibody binding was blocked by incubation with 0.5% (*w/v*) BSA in PBS-T (0.05% (*v/v*) Tween 20 in PBS) buffer for 2 h at 37 °C. After incubation, the plates were washed and mouse sera were tested by adding five serial dilutions of each serum and standard monoclonal anti-OVA IgG preparations in duplicates. Plates were incubated overnight at room temperature, washed, and analyzed for OVA-specific IgG levels by incubation with HRP-conjugated goat anti-mouse IgG (2 h at 37 °C). After washing, plates were incubated with 0.6 mg/mL *o*-phenylenediamine dihydrochloride solution in citrate phosphate buffer, pH 5.0, with 30% H_2_O_2_ (0.5 μL/mL) for 30 min at room temperature in the dark. The enzymatic reaction was stopped with 12.5% H_2_SO_4_. Absorbance at 492 nm was measured using a microplate reader (Thermo Fisher Scientific, Waltham, MA, USA). For quantification of OVA-specific immunoglobulin G subclasses, IgG1 and IgG2a, plates were coated with OVA and incubated with sera samples as described above. After washing, biotinylated rat anti-mouse IgG1 or IgG2a was added and incubated for 2 h at 37 °C and subsequently with the streptavidin-HRP conjugate for another 2 h at 37 °C. After washing, the substrate solution was added and incubated in the dark for 30 min at RT. The enzyme reaction was stopped with H_2_SO_4_ (12.5% (*v/v*)) and absorbance at 492 nm was measured using a microplate reader (Bio-Rad Microplate Reader). The relative quantities of anti-OVA IgGs were calculated by parallel line assays. Each serum was compared with standard monoclonal anti-OVA IgG, anti-OVA IgG1, and anti-OVA IgG2a preparations.

### 2.7. Statistics

Data analysis was performed using Prism software (version 9; GraphPad Software, San Diego, CA, USA). Statistical significance was determined by one-way ANOVA followed by Dunnett’s multiple comparisons test. A *p* value < 0.05 was considered statistically significant.

## 3. Results and Discussion

### 3.1. Characterization of Liposomes Incorporating SG29, SG115, and ZSB63

The modified lipid-film hydration method was used to prepare the liposomal formulations of SG29, ZSB63, and SG115. The obtained liposomal formulations were then characterized by DLS in terms of size distribution, polydispersity index, and zeta potential (see Table 2). The entrapment efficiency of SG29, SG115, and ZSB63 was determined by HPLC and evidently all compounds were incorporated in the liposomes at a high yield surpassing 97% (Table S3 (Supplementary Materials)). The high entrapment efficiency is probably related to the chemical structure and lipophilicity of the tested compounds; the calculated clogP values are as follows: SG29, clogP = 3.773; SG115, clogP = 12.143; ZSB63, clogP = 7.997. The incorporation of all three examined compounds into liposomes brought about a significant and approximately two-fold increase in the size of liposomes (hydrodynamic diameters) when compared to that of the empty liposomes (166 nm). The average size of compound-loaded liposomes was as follows: 414 nm (SG29); 400 nm (ZSB63); and 304 nm (SG115). The adjuvant activity has been shown to depend on the size of liposomes [28]. The latter plays a significant role in the type of generated immune response. For example, liposomes larger than 225 nm have exhibited a Th1-biased, and those smaller than 155 nm a Th2-biased, response [29]. Similarly, it was reported that immunization with small size (100 nm) liposomes produces a Th2 type response, while immunization with large size (≥400 nm) liposomes generates a predominantly Th1-skewed immune response [30]. Interestingly, Lopes et al. found that the entrapment of small amphiphilic molecules caused a decrease in liposome size possibly due to incorporated drug–lipid interaction [31]. This was not the case with our desmuramylpeptides, which can be considered somewhat amphiphilic. On the contrary, a significant increase in liposome size with all incorporated examined desmuramylpeptides was found. The increase in liposome size with incorporated compounds SG29 and ZSB63 was most pronounced, while the liposomes with incorporated compound SG115 carrying a stearyl group as a lipophilic anchor, were also increased compared to empty liposomes, but to a lesser extent. The observed changes in size could be ascribed to the chemical properties of examined compounds that affected their accommodation inside the liposomes and the interaction of lipid bilayers and incorporated compounds but further studies are needed in order to more precisely clarify the interactions of the lipid bilayer and incorporated compounds. We have shown previously that structurally related adamantyl tripeptides have a great capacity to interact with lipids in negatively charged liposomes [26,32]. The size distribution was sharp and the polydispersity index was about 0.44. The PdI values of liposomes with ZSB63 (0.45) and SG29 (0.43), in particular, were comparable to those of empty liposomes (0.35), thus indicating a normal width of size distribution and good homogeneity of the generated dispersions. In the case of SG115-loaded liposomes, the PdI was slightly increased to 0.59 but still within the limits of normal size distribution. The zeta potential is related to the charge on the surface of the particles and is important for the interactions of liposomes with biological components in an in vivo setting. According to a previously published report [33], the negative charge of liposomes favors the interaction with the reticuloendothelial systems and stabilizes liposome dispersion. The measured zeta potential values of the examined liposomal formulations were comparable (−3.50 for SG29 and −4.53 for SG115; −3.93 for empty liposomes) but there was a small difference for liposomes loaded with compound ZSB63. Since the zeta potential reflects the surface charge of liposomes, it may be assumed that the observed differences are probably directly related to the interaction of the incorporated compounds with the polar head of the phospholipid molecules in the lipid bilayer as well as to the modification of the overall structural characteristics of the lipid bilayer [34]. Namely, the SG115 compound contains a stearoyl moiety, which is deeply embedded in the lipophilic part of the bilayer while the amphiphilic adamantane-featuring derivative ZSB63 and parent compound SG29 can also alter the structure of the bilayer in some fashion. 

### 3.2. In Vivo Adjuvant Properties of SG29, SG115, and ZSB63 in OVA-Induced Antibody Responses

**Figure 1:**
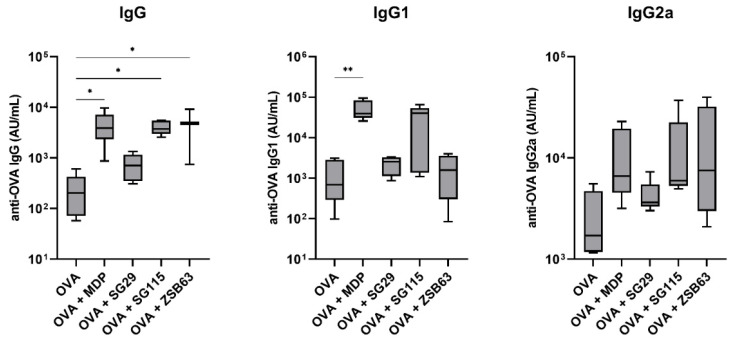
Ovalbumin-specific IgG (**left**), IgG1 (**middle**), and IgG2a (**right**) responses in NIH/OlaHsd mice after immunization with OVA-loaded neutral liposomes (10 μg OVA per dose), adjuvanted with MDP or compounds SG29, SG115, and ZSB63 (0.30 μmol adjuvant per dose). The concentrations were measured one week after the booster dose. Data are means ± SEM of 5 mice per group. *, *p* < 0.05; **, *p* < 0.001.

Lipophilic MDP derivatives have shown a tendency to promote a Th1-biased cellular immune response, especially when used in conjunction with liposomes. To determine if and how the introduction of specific lipophilic moieties into the structure of compound SG29 affects the in vivo adjuvant activity, an immunization study was conducted in a murine model of adjuvanticity. Specifically, SG29 and its lipidated analogs SG115 and ZSB63 were investigated for the induction of systemic immune responses against the model antigen OVA. Five groups of NIH/OlaHsd mice were immunized with OVA-containing neutral liposomes, either alone or additionally adjuvanted with our positive control MDP or the desmuramylpeptides SG29, SG115, and ZSB63. After the second booster dose, the mice sera were collected and examined for OVA-specific IgG antibodies (Figure 1). 

As expected, due to the low immunogenicity of OVA, its liposomal formulation without added adjuvants induced weak systemic responses and therefore served as the negative control. In agreement with previous experiments, MDP as the positive control significantly enhanced the production of anti-OVA IgG antibodies (19.1-fold), while SG29 showed only marginal adjuvant activity. On the other hand, a significant boost in the elicited total IgG responses was also seen in the group immunized with the addition of the adamantane-carrying derivative ZSB63 (20.4-fold), followed by a 17.3-fold boost by SG115, featuring a C18 lipophilic tail on the aromatic ring, while SG29 had a marginal effect on the total IgG levels. To interrogate the nature of the induced immune responses with regard to Th1/Th2 polarization, the levels of the Th1-associated IgG1 and Th2-associated IgG2a antibody isotypes were also measured (Figure 1). In most experimental groups, the levels of induced anti-OVA IgG1 closely resembled the total IgG levels. MDP and SG115 highly amplified the production of IgG1, while the adamantane-featuring ZSB63 only induced a marginal increase in IgG1 levels, comparable to that of SG29. On the other hand, there were notable differences in the enhancement of IgG2a production, which altered the Th1/Th2 bias of the provoked immune responses (see Table 3). Consistent with previous reports, MDP induced a predominantly Th2-biased response, characterized by significant increases in IgG1 generation, and less pronounced increases in IgG2a, compared to the unadjuvanted control. Likewise, a predominantly IgG1-based response was elicited by SG115, while in the group immunized by ZSB63, a marked increase in the IgG2a response was observed, which indicated a shift toward the Th1 response.

Interestingly, while the adamantane-based ZSB63 and SG29 had comparable potencies in vitro, the latter was devoid of adjuvant activity under the employed conditions. This observation contradicts the previous report of excellent in vitro/in vivo correlation demonstrated for NOD2 agonists by Rubino et al. [35]. An analogous discrepancy was noticeable with SG115, which albeit displayed only weak in vitro NOD2 activating capacity in the micromolar range, showed in vivo adjuvant activity comparable to those of MDP and ZSB63. This is in agreement with our previous findings that the addition of the C18 fatty acid chain converts an in vitro weakly active NOD2 agonist into a potent in vivo adjuvant [8]. It is evident that the in vitro and in vivo activities of the desmuramylpeptide NOD2 agonists do not correlate in a linear fashion. Given that SG29, SG115, and ZSB63 are all prodrugs of the same active compound, their disparate effects on the induction of humoral immune responses probably originate from their distinctive physicochemical properties.

## 4. Conclusions

In the present study, we interrogated how the decoration of an in vitro active NOD2 agonist SG29 with different lipophilic moieties affects the in vivo adjuvant activity. The data show that the introduction of a C18 or an adamantane lipophilic anchor into the structure of SG29, to produce SG115 and ZSB63, respectively, substantially improved the in vivo adjuvant activity, which can be attributed to a better anchoring in the liposomal lipid bilayer. These results are consistent with previous studies that indicate an important relationship between NOD2 activity and the ability of compounds to localize to the membrane. The incorporation of the adamantane group potently enhanced the total IgG levels but mostly augmented the production of IgG2a antibodies, which indicated a shift toward a Th1 immune response, while the attachment of stearoyl moiety produced a Th2-biased response, similar to that of MDP. Given that the majority of the currently used adjuvants induce predominantly Th2-biased responses, the induction of a Th1-biased response still constitutes a highly sought-after trait. The bona fide capacity of ZSB63 to guide the type of response towards a potent cellular (Th1) response therefore highlights its untapped potential as an alternative vaccine adjuvant.

## Figures and Tables

**Table 1:** NOD2 agonistic activities of lipidated SG29 analogs.

Compound	Structure	NOD2 EC_50_ ^a^
SG29	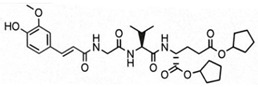	45 ± 6 nM
SG115	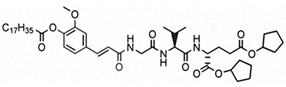	6.2 ± 1.2 µM
ZSB63	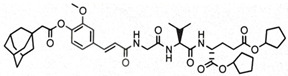	44 ± 9 nM

^a^ SEAP activities were measured in NOD2-specific HEK-Blue cell supernatants after incubation for 18 h; EC50 values are means ± SEM of at least two independent experiments with 7 or 8 concentrations used (from 1 nM to 20 µM).

**Table 2:** Size, polydispersity index, and zeta potential of the examined liposomal formulations.

Sample	Z-Average (nm)	PdI	ZP (mV)
Liposome	166.4 ± 10.83	0.35 ± 0.07	−3.93 ± 0.58
Liposome + SG29	413.53 ± 25.72	0.43 ± 0.06	−3.50 ± 0.71
Liposome + SG115	303.94 ± 14.38	0.59 ± 0.1	−4.53 ± 0.72
Liposome + ZSB63	399.65 ± 9.15	0.45 ±0.01	−2.88 ± 0.32

**Table 3:** The ratio of anti-OVA IgG1 and anti-OVA IgG2a levels following a second booster.

Experimental Group	Log10 (Anti-OVA IgG1/Anti-OVA Igg2a) ^1^
OVA in liposomes	−0.580 ± 0.264
(OVA + MDP) in liposomes	0.747 ± 0.171
(OVA + SG29) in liposomes	−0.325 ± 0.161
(OVA + SG115) in liposomes	0.120 ± 0.386
(OVA + ZSB63) in liposomes	−0.935 ± 0.393

^1^ The IgG1/IgG2a ratio was calculated after the second booster for each mouse serum. The results for each experimental group (*n* = 5; females) are reported as the means ± SEM.

## Data Availability

Data are contained within the article.

## Supplementary Materials

The following supporting information can be downloaded at: https://www.mdpi.com/article/10.3390/pharmaceutics14122755/s1, Table S1: HPLC gradient system for compounds SG29 and ZSB63, flow rate 1.0 mL/min; Table S2: HPLC gradient system for compound SG115, flow rate 1.23 mL/min; Table S3: The entrapment efficiency of examined compounds in liposomes; Figure S1: The chromatogram of compound SG29 (left) and chromatogram of supernatant after separation of liposome pellet with incorporated compound SG29 (right); Figure S2: The chromatogram of compound SG115 (left) and chromatogram of supernatant after separation of liposome pellet with incorporated compound SG115 (right); Figure S3: The chromatogram of compound ZSB63 (left) and chromatogram of supernatant after separation of liposome pellet with incorporated compound ZSB63 (right); Figure S4: Standard curves of compounds SG115 (left) and SG29 (right) used for entrapment efficiency calculation.
