# Supplementary material for: Lipidation of NOD2 Agonists with Adamantane and Stearoyl Moieties Differentially Regulates Their In Vivo Adjuvant Activity
Source: Pharmaceutics. 2022 Dec 9;14(12):2755. doi: 10.3390/pharmaceutics14122755 (PMC9785857; doi:10.3390/pharmaceutics14122755)
Supplement: Supplementary file 1 [file pharmaceutics-14-02755-s001.zip › pharmaceutics-1997883-supplementary.pdf]

SUPPLEMENTARY MATERIALS

Lipidation Of NOD2 Agonists with Adamantane And Stearoyl Moieties Differentially Regulates Their In Vivo Adjuvant Activity

Samo Guzelj,[1†] Marcela Šišić,[2†] Špela Bizjak,[1] Leo Frkanec,[3] Ruža Frkanec*,[2] Žiga Jakopin*,[1]

[1]Faculty of Pharmacy, University of Ljubljana, SI-1000 Ljubljana, Slovenia

[2]Centre for Research and Knowledge Transfer in Biotechnology, University of Zagreb, 10000 Zagreb, [3]Institut Rudjer Bošković, Bijenička St. 54, 10000 Zagreb, Croatia

*Corresponding author

Ruža Frkanec

University of Zagreb

Centre for Research and Knowledge Transfer in Biotechnology

Rockefeller St. 10

1010000 Zagreb, Croatia

Email:rfrkanec@unizg.hr

Entrapment efficiency determination by HPLC

Chromatographic separations were carried out using Waters 2695 Alliance separations module, Waters 2996 PAD detector, and Waters Symmetry C18 5 μm, 4.6 x 250 mm column. All analyses were performed at 40 °C. The percentage of each peak in the respective chromatograms was calculated by the integration of the UV response (peak area) at 214 nm. Two HPLC methods, using a gradient solvent system made of acetonitrile and water containing 0.1 % TFA have been developed. The developed methods and corresponding gradient solvent systems differed in the run time of analysis, the flow rate of mobile phase, and acetonitrile/water rate change over run time, as indicated in Table 1 and Table 2. All standard samples were dissolved in acetonitrile and filtered through a 0.45 μm PVDF filter (Hydrophilic PVDF 0.45 μm, Millipore Millex-HV) before analysis.

In order to determine the encapsulation efficiency of examined compounds, the liposome suspensions of compounds were placed into centrifugation tubes and centrifuged in Eppendorf centrifuge 5810 R for one hour at 22 000 rpm. After centrifugation, the supernatants were separated from the pellets, filtered through a 0.45 μm PVDF filter, and submitted to HPLC analyses.

The entrapment efficiency was calculated based on a previously constructed standard curve for each of the tested compounds. The amounts of the nonentrapped compounds were determined using a standard curve and the amounts of the entrapped compounds were calculated by subtracting the obtained value from the total amount of the compound used for liposome preparation. The results are presented in Table 3.

Table S1. HPLC gradient system for compounds SG29 and ZSB63, flow rate 1.0 mL/min.

t / min	% A*	% B
0	75	25
20	5	95
40	5	95
45	75	25

*Solvents: A – H_2O + 0,1% TFA; B – ACN

Table S2. HPLC gradient system for compound SG115, flow rate 1.23 mL/min.

t / min	% A	% B
0	80	20
15	5	95
40	5	95
55	80	20

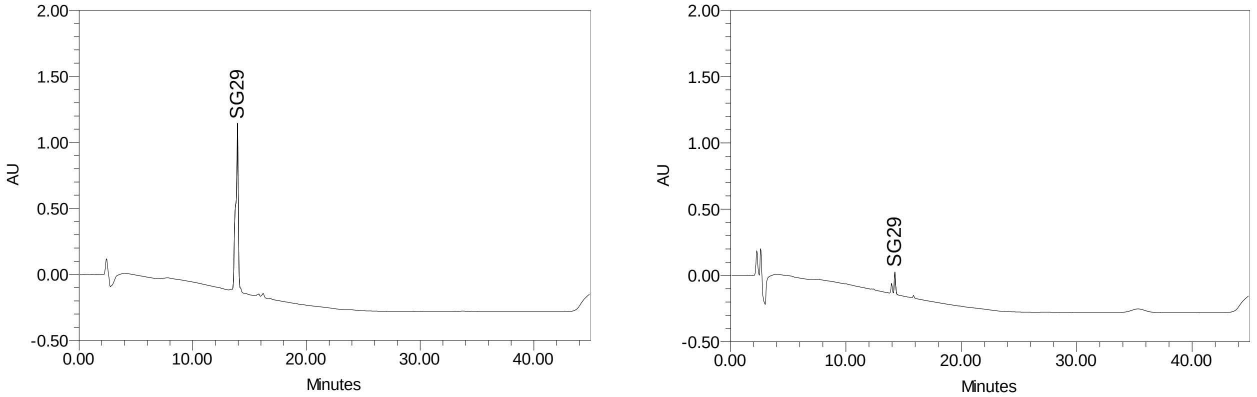

Figure S1. The chromatogram of compound SG29 (left) and chromatogram of supernatant after separation of liposome pellet with incorporated compound SG29 (right).

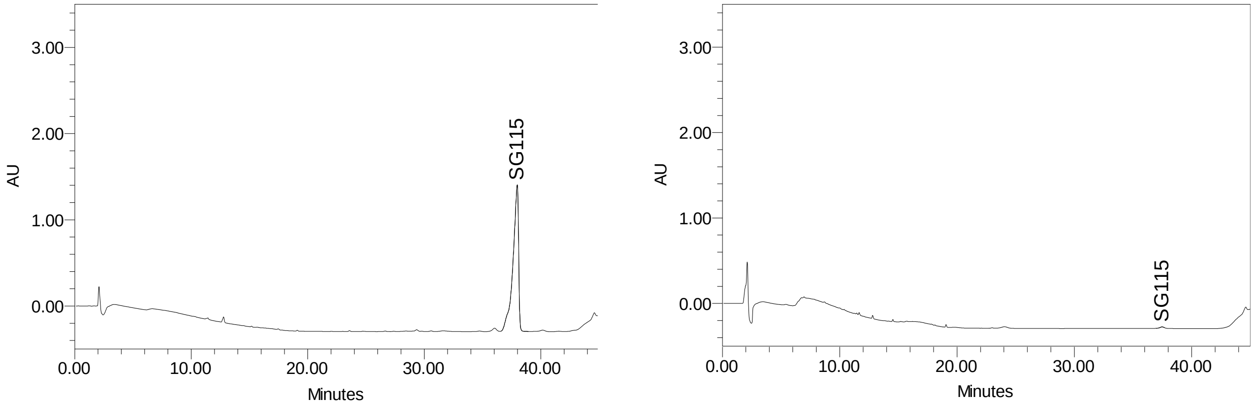

Figure S2. The chromatogram of compound SG115 (left) and chromatogram of supernatant after separation of liposome pellet with incorporated compound SG115 (right**).**

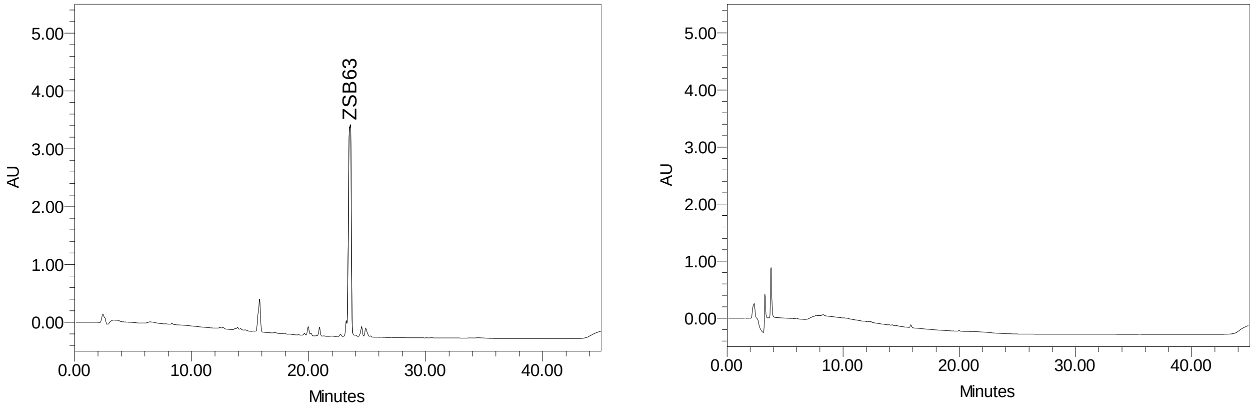

Figure S3. The chromatogram of compound ZSB63 (left) and chromatogram of supernatant after separation of liposome pellet with incorporated compound ZSB63 (right).

Table S3. The entrapment efficiency of examined compounds in liposomes*.

Compounds	Entrapment efficiency (%)
ZSB63	~100
SG115	98.5 ± 0.19
SG29	97.6 ± 0.29

* the results are expressed as an average value ± standard deviation (SD) of three separate experiments

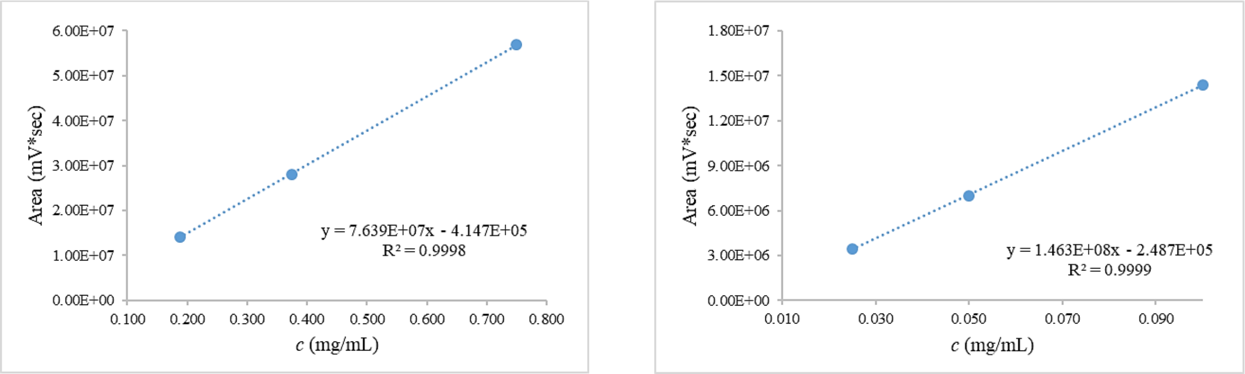

Figure S4. Standard curves of compounds SG115 (left) and SG29 (right) used for entrapment efficiency calculation
